# On-pump versus off-pump coronary artery bypass surgery in patients older than 60 years: five-year follow-up of MASS III trial

**DOI:** 10.1186/1749-8090-9-136

**Published:** 2014-08-05

**Authors:** Rodrigo Morel Vieira de Melo, Whady Hueb, Paulo Cury Rezende, Eduardo Gomes Lima, Alexandre Ciappina Hueb, José Antonio Franchini Ramires, Roberto Kalil Filho

**Affiliations:** Heart Institute (InCor) University of Sao Paulo, Sao Paulo, Brazil; Department of Atherosclerosis, Heart Institute of the University of Sao Paulo, Sao Paulo, Brazil; Av. Dr. Eneas de Carvalho Aguiar 44, AB, Sala 114, Cerqueira César, CEP 05403-000 São Paulo, SP Brazil; Hospital Alemao Oswaldo Cruz, Sao Paulo, Brazil

**Keywords:** Coronary artery disease, Coronary artery bypass grafts, CABG, Cardiopulmonary bypass, CPB

## Abstract

**Background:**

We aim to evaluate in-hospital events and long-term clinical outcomes in patients over 60 years of age with stable coronary artery disease and preserved left ventricular ejection fraction undergoing off-pump or on-pump coronary artery bypass grafting.

**Methods:**

The MASS III was a single-center randomized trial that evaluate 308 patients with stable coronary artery disease and preserved ventricular function assigned for: 155 to off-pump and 153 to on-pump CABG. Of this, 176 (58.3%) patients were 60 years or older at the time of randomization (90 of-pump and 86 on-pump). The primary short-term end point was a composite of myocardial infarction, stroke, and overall mortality occurring within 30 days after surgery or before discharge, whichever was later. The primary long-term end point was death from any cause within 5 years, non-fatal myocardial infarction between 30 days and 5 years, or additional revascularization between 30 days and 5 years.

**Results:**

On-pump CABG had a higher incidence of 30-day composite outcome than off-pump CABG (15,1% and 5.6%, respectively; *P* = 0.036). However, after the multivariate analysis, this association lost statistical significance, *P* = 0.05. After 5-year follow-up, there were no significant differences between both strategies of CABG in the composite end points 16.7% and 15.1%; Hazard Ratio 1.07; CI 0.41 – 1.82; *P* = 0.71, for off-pump and on-pump CABG respectively.

**Conclusions:**

On-pump and off-pump CABG achieved similar results of combined events at short-term and 5-year follow-up.

**Trial registration:**

Clinical Trial Registration Information—URL: http://www.controlled-trials.com. Registration number: ISRCTN59539154.

## Background

Several studies comparing off-pump coronary artery bypass surgery (CABG) with on-pump CABG, in various patients populations, failure to demonstrate a superiority of a technique over the other in clinical outcomes despite some differences in postoperative complications [1–3]. Currently, there is an attempt to identify patient subgroups in whom beating heart surgery is the preferred procedure, particularly in those with a high risk profile [4–6].

Advanced age is associated with a higher prevalence of preoperative comorbidities, reduced functional reserve, and increased mortality and morbidity in patients undergoing coronary artery bypass grafting [7, 8]. That becomes a major concern as the number of elderly patients undergoing CABG surgery continues to increase [9, 10]. Thus, recent trials addressed for this specific population, did not include data on long-term morbidity and mortality [5, 6].

The MASS III was a randomized clinical trial that evaluated postoperative outcomes and long-term clinical events in patients with multivessel coronary artery disease, stable angina, and preserved ventricular function, undergoing coronary artery bypass grafting with and without extracorporeal circulation [3].

The main finding of the MASS III trial was that either revascularization strategy provides similar rates of all-cause mortality and major cardiovascular events at 5 years. The present post-hoc analysis aims to investigate whether off-pump CABG strategy would be especially advantageous for older patients undergoing CABG on postoperatively outcomes and long-term follow-up.

## Methods

### Study design and treatment

Protocol details have been published previously [11]. In brief, patients with angiographically documented proximal multivessel coronary stenosis of >70% by visual assessment, stable angina, and preserved ventricular function were considered for inclusion. Patients were enrolled and randomized if the surgeons agreed that revascularization could be achieved by either strategy. All angiograms were reviewed, and a surgical plan was documented before randomization. Patients were eligible if they were referred for isolated coronary bypass surgery for the first time and an off-pump procedure was deemed technically feasible. In this post-hoc analysis, we included only patients with 60 years or older at the time of randomization. Patients were excluded if they required emergency or concomitant major surgery, unstable angina requiring emergency revascularization, ventricular aneurysm requiring repair, and a left ventricular ejection fraction of less than 40%, previous stroke, peripheral vascular disease or chronic renal insufficiency with a estimated creatinine clearance of less than 60 mL/min. Patients were also excluded if they were unable to provide written informed consent. The Ethics Committee of the Heart Institute of the University of São Paulo - Medical School, in São Paulo Brazil, approved the trial, and all procedures were performed in accordance with the Helsinki Declaration. All subjects gave informed consent. Trial operators were required to perform optimum coronary revascularization in accordance with current best practices. The procedure was performed by surgeons experienced in both on-pump and off-pump bypass surgery. Stabilization devices were used during off-pump surgery to allow the safe construction of the anastomosis of the graft with the recipient artery.

### Qualification of surgeons

Each operation was performed by a surgeon with more than 20 years of experience and having completed more than 100 procedures per year in both techniques.

### Study end points

The primary short-term end point was a composite of myocardial infarction, stroke, and overall mortality occurring within 30 days after surgery or before discharge, whichever was later. The primary composite long-term end point was death from any cause within 5 years, non-fatal myocardial infarction between 30 days and 5 years, or additional revascularization between 30 days and 5 years. Stroke was defined as a focal brain injury that persisted for >24 hours, combined with an increase in disability of at least 1 grade on the Ranking scale [12]. Myocardial infarction within 7 days from the coronary artery bypass grafting procedure was considered if elevation of CK-MB or troponin 5 times or more the 99^th^ percentile.

### Follow-up

Adverse and other clinical events were tracked from randomization. Patients were assessed with follow-up visits every 6 months at the Heart Institute.

### Statistical analysis

All data were analyzed on an intention-to-treat principle beginning immediately after randomization. The risk of an event after on-pump surgery was compared with that after off-pump surgery, and the results are presented as the absolute difference with the corresponding 95% confidence intervals. Values are expressed as mean (±SD). Dichotomous data were compared by the χ^2^statistic or Fisher exact test. Continuous variables that were not distributed normally, as evaluated through the Kolmogorov–Smirnov test, were compared by the Mann–Whitney test. Continuous variables with a normal distribution were compared by the Student’s t test. All reported probability values are 2-sided. Event-free survival was graphically compared by using Kaplan–Meier curves. Event rates were compared with the use of the log-rank test of time to the first event after randomization. Relative risks were expressed as hazard ratios with associated confidence intervals and were derived from the Cox proportional-hazards model. A probability value of *P* < 0.05 was considered statistically significant. Multivariable analysis with logistic regression (short-term end point) and Cox proportional-hazards model (long-term end point) were used when appropriated, including variables with a possible association (*P* < 0.2) with the combined events. These analyses were performed with SPSS, version 17.0 (SPSS, Inc).

## Results

Between March 2001 and March 2006, 308 patients with stable coronary artery disease were assigned to CABG: 153 to on-pump surgery and 155 to off-pump surgery. From those, 176 (58.3%) patients were 60 years or older at the time of randomization (90 off-pump CABG and 86 on-pump CABG). The two groups were well-matched for baseline demographic, clinical, and angiographic characteristics and are summarized in Table 1. The mean age was 65.7 in the on-pump patients and 67.1 in the off-pump group. There was no crossover between study groups and there was no lost of follow-up. The median follow-up was 5 years.

**Table 1:** **Baseline characteristics**

	On-pump CABG n = 86	Off-pump CABG n = 90	***P***
Age (years)	66.5 ± 5.1	67.9 ± 5.1	0.07
Male gender n (%)	64 (74.4)	62 (68.9)	0.50
Diabetes n (%)	32 (37.2)	29 (32.2)	0.029
Hypertension	58 (67.4)	61 (67.8)	1.0
Current smoke n (%)	17 (19.8)	14 (15.6)	0.74
Previous MI n (%)	35 (40.7)	44 (48.9)	0.29
Angina n (%)	81 (94.2)	84 (93.3)	1.0
Angina CCS III-IV n (%)	10 (12.3)	15 (17.9)	0.38
Ejection fraction	65.1 ± 6.4	65.8 ± 9.3	0.57
Cholesterol mg/dL	213 ± 48.6	211 ± 40.9	0.74
LDL-c mg/dL	134 ± 45.1	132 ± 34.8	0.68
HDL-c mg/dL	42 ± 9.2	42 ± 10.2	0.82
Triglycerides mg/dL	175 ± 122	187 ± 111	0.52
3-Vessel CAD n (%)	73 (84.9)	67 (74.4)	0.09

CABG = coronary artery bypass graft; MI = myocardial infarction; CCS = Canadian Cardiovascular Society; CAD = coronary artery disease.

Patients randomized to on-pump CABG were more likely to achieve complete revascularization than off-pump CABG with a non-significant trend, (52.3%) vs (38.9%) p = 0.096, respectively. There was no difference in the amount of grafts of left internal thoracic artery in the two revascularization strategies: 81 (94.2%) vs 88 (97.8%) for on-pump and off-pump CABG respectively (*P* = 0.27). Moreover, patients undergoing on-pump surgery showed a higher average total number of grafts 2.9 ± 0.64 vs 2.6 ± 0.61, *P* = 0.002 and a higher number of vessels treated 3.0 ± 0.71 vs 2.7 ± 0.68, *P* = 0.001.

On the short-term end point, the univariate analysis demonstrated that on-pump CABG patients had a higher incidence of combined events before discharge or within 30 days after the procedure as compared with off-pump CABG: 13 (15.1%) vs 5 (5.6%); *P* = 0,04 (Table 2) (Table 3). After the multivariate analysis, with the inclusion of the following variables: on-pump surgery, current smoke and age, performing on-pump CABG showed a trend for association with the short-term combined end point, p = 0.05.

**Table 2:** **Univariate predictors of short**-**term combined events**

	With combined events n = 18	Without combined events n = 158	***P***
On-pump CABG n (%)	13 (72.2)	73 (46.2)	0.04
Age, years	65.3 ± 4.2	67.4 ± 5.2	0.10
Male gender n (%)	11 (61.1)	115 (72.8)	0.41
Hypertension n (%)	14 (77.8)	105 (66.5)	0.43
Diabetes n (%)	4 (22.2)	57 (36.1)	0.30
Current smoke n (%)	6 (33.3)	25 (15.8)	0.10
Angina n (%)	11 (61.1)	115 (72.8)	0.41
3-Vessel CAD n (%)	16 (88.9)	124 (78.5)	0.37
Previous MI n (%)	17 (94.4)	148 (93.7)	1.0
Grafts, mean	2.9 ± 0.58	2.7 ± 0.65	0.25
Treated vessels, mean	3 ± 0.59	2.8 ± 0.74	0.38
LIMA graft n (%)	17 (94.4)	152 (96.2)	0.53

CABG = coronary artery bypass graft; MI = myocardial infarction; CAD = coronary artery disease; LIMA = Left Internal Mammary Artery.

**Table 3:** **Events at 30 days**

	On-pump CABG	Off-pump CABG	***P***
Myocardial infarction n (%)	10 (11.6)	4 (4.4)	0.09
Stroke n (%)	3 (4.4)	1 (1.1)	0.36
Death n (%)	0	0	-
Combined events n (%)	13 (15.1)	5 (5.6)	0.04

CABG = coronary artery bypass graft.

**Figure 1:**
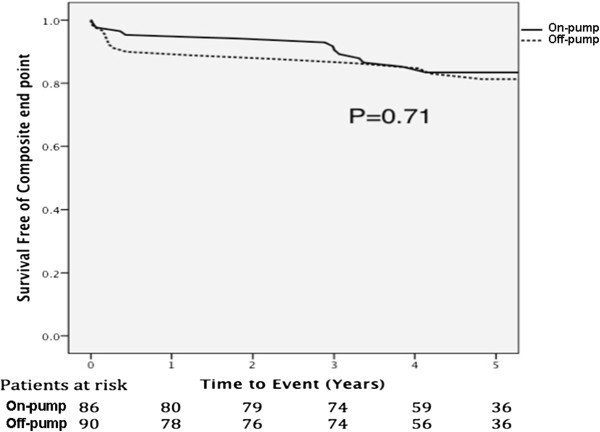
**Kaplan–** **Meier estimates of survival free of combined events after surgery.**

After 5-year follow-up, on the univariate analysis, there were no significant differences between both strategies of CABG in the composite end points 15 (16.7%) vs 13 (15.1%), (Hazard Ratio 1.07; CI 0.41 – 1.82; *P* = 0.71) for off-pump and on-pump CABG respectively (Figure 1).

## Discussion

There is currently an attempt to compare clinical outcomes in the two techniques of myocardial revascularization, on short and long-term follow-up. So far, previous studies have either underrepresented an elderly high-risk population or had lack of data on long-term follow-up.

In this post hoc analysis, we seek to evaluate a population at higher surgical risk selected by having over 60 years old at the time of randomization, and assess whether off-pump CABG add clinical benefit specifically on 5-year follow-up.

In the Rooby Trial, with follow-up results available so far 1 year, both techniques had similar clinical events at 30 days but with a lower rate of combined events at 1 year favoring the on-pump group [1].

The CORONARY study aimed to compare the two techniques in a population of greater surgical risk (older patients with a higher cardiovascular risk), showing no difference in clinical events at 30 days and 1-year follow-up [2].

The MASS-3 was a randomized, single-center study that evaluated two strategies for elective CABG in patients with stable coronary artery disease and preserved ventricular function. The main result was that there was no difference in the combined end point between the two groups following 5 years [3].

Specifically in the population with advanced age, the recent studies GOPCABE and DOORS, showed no difference in clinical events on short and intermediate follow-up [5, 6].

Despite being a post-hoc analysis in a specific population of the original trial, both study groups had well-balanced baseline characteristics. Most of our patients had 3-vessel CAD and complete revascularization was the goal in both groups, but like previous studies, off-pump surgery was associated with a lower proportion of complete revascularization. This was evidenced by a lower average in total number of grafts and number of vessels treated on the off-pump group.

In the 30-day analysis, patients undergoing off-pump surgery had a lower incidence of combined events on the univariate analysis, driven by a lower rate of stroke and myocardial infarction. However, after the multivariate model, this analysis lost statistical significance revealing only a trend towards association.

There is some contradictory data in the literature assessing however off-pump CABG in patients with advanced age can significantly reduce rates of perioperative overall complications. Observational studies have suggested that off-pump technique might prevent stroke and postoperative myocardial infarction [13–17]. However, more recent trials did not confirm any benefit for any technique over the other on rates of in-hospital complications [5, 6].

The results of 5-year follow-up confirm the data obtained in previous studies with short to intermediate follow-up that both techniques are safe and similar in terms of clinical events in patients with advanced age.

The strength of this study lies on the long-term follow-up in a population with a higher surgical risk, with no loss to follow-up.

There were some limitations. First, we used the cut-off level of troponin and CK-MB for the diagnosis of myocardial infarction after the procedure of 5 times the 99th percentile, consistent with the definition used at the time of the study, which is a lower limit than current recommendations of the third definition of myocardial infarction [18]. Although we selected only patients older than 60 years at the time of randomization, the average age of our patients was not very high. There was only assessment of clinical outcomes of our patients, as graft patency and neurocognitive evaluation were not assessed in this study. In addition, there is no data concerning stenosis of carotid artery and cerebrovascular history for comparison between the two groups. Finally, this post-hoc analysis was not pre-specified in the original design of the MASS III Trial and therefore, subject to the biases inherent of this analysis.

## Conclusion

In our study, patients older than 60 years undergoing coronary surgical revascularization achieved similar rates of composite end points on short-term and long-term follow-up with on-pump and off-pump CABG.
